# Evaluation of sperm recovered after slaughter from cauda epididymides of red Sokoto bucks

**DOI:** 10.14202/vetworld.2016.1440-1444

**Published:** 2016-12-18

**Authors:** A. H. Abu, A. I. Kisani, T. Ahemen

**Affiliations:** 1 Department of Veterinary Physiology, Pharmacology and Biochemistry, College of Veterinary Medicine, Federal University of Agriculture, P.M.B. 2373, Makurdi, Benue State, Nigeria; 2 Department of Veterinary Surgery and Theriogenology, College of Veterinary Medicine, Federal University of Agriculture, P.M.B. 2373, Makurdi, Benue State, Nigeria; 3 Department of Animal Breeding and Physiology, College of Animal Science, Federal University of Agriculture, P.M.B. 2373, Makurdi, Benue State, Nigeria

**Keywords:** epididymides, goat bucks, spermatozoa, testes

## Abstract

**Aim:**

Viable spermatozoa could be recovered from the cauda epididymides for the purpose of preservation of genetic material of male animals with desirable traits and for use in reproductive biotechnology. The aim of this study was to determine the effect of storage time on testicular and epididymal biometry, sperm reserves and epididymal sperm characteristics of red Sokoto bucks post mortem.

**Materials and Methods:**

Testes-epididymides were collected immediately after slaughter of mature red Sokoto bucks and transported in ice chest to the laboratory. The samples were either processed immediately or stored at 5°C in refrigerator for 24, 48 h and then processed. The testes and epididymides were measured and weighed. Sperm motility, concentration, livability, morphology, intact acrosome, and sperm reserves from different treatment groups including control were evaluated and means (±standard error of mean) were recorded.

**Results:**

There was no significant difference (p>0.05) in the testicular and epididymal dimensions determined between the means of the groups. Percent sperm motility and viability decreased significantly (p<0.05) after 24 h from 69.00±0.46 and 71.27±0.50% to 50.60±0.48 and 60.47±0.70% at 48 h, respectively. Significant decreases (p<0.05) in epididymal sperm concentration and intact acrosome from 2.86±0.08 and 92.87±0.39 at 0 to 24 h of storage, respectively, were observed.

**Conclusion:**

The results of this study suggest that spermatozoa recovered from the epididymides of red Sokoto bucks were viable after storage for up to 48 h. Furthermore, this finding offers some hope that epididymal sperm recovered post-mortem can be used in assisted reproductive technologies.

## Introduction

In the field of animal reproduction, artificial insemination and sperm cryopreservation are methods of reproductive biotechnologies that have revolutionized the livestock industry. Sperm recovered from cauda epididymides can be used in the conservation of animal genetic material and reproductive biotechnology. However, any plan of action for *ex situ* conservation of animal genetic material of valuable and or endangered male animals requires protocols for the recovery of viable sperm from the epididymides of slaughtered animals.

Instead of the conventional method of semen collection, epididymal sperm is rather collected to reduce costs and because of some organizational difficulties. This becomes necessary when natural mating or the use of ejaculated semen is not possible due to difficulty of handling intractable animal or sudden death of an animal [1-3]. Acceptable motility and viability of spermatozoa recovered from the epididymides which have been maintained at room temperature or 5°C has been reported in bull [4], boar [5], stallion [6], tom cat [7], and dog [8]. Researchers have reported that the method used to recover sperm from cauda epididymides postmortem does not affect their quality [9]. However, other studies have recorded that quality of epididymal spermatozoa varied according to climactic conditions and temperature [10].

Preservation of sperm cells can be compromised if testis-epididymis samples are poorly packaged or stored due to post-mortem tissue degeneration [11]. Furthermore, time constraint is consistently identified as a major problem for recovering motile and viable epididymal sperm cells from the abattoir-derived testicles and epididymides [5,12]. A Google search through available literature showed a lack of wealth of information on the effect of handling conditions or storage time on the quality of spermatozoa recovered post-mortem from cauda epididymides of local breeds of goats in Nigeria.

The aim of this study was to determine the effect of storage time on testicular and epididymal biometry, and epididymal sperm characteristics of red Sokoto bucks post-mortem.

## Materials and Methods

### Ethical approval

An ethical approval was not necessary since no live animals were used for this research. However, the samples were collected from a metropolitan abattoir approved by the Benue State Government of Nigeria.

### Location of the study

This study was conducted at the College of Veterinary Medicine, Federal University of Agriculture, Makurdi, Nigeria. Makurdi is situated in the Southern Guinea Savannah and located at Latitude 7°14’ North and Longitude 8°21’ East. The area is warm with temperature range of 24-36°C and high temperature is experienced between late February and April. The rainfall is between 508 and 1016 mm. There are two seasons: Rainy season (May-October) and dry season (November-April).

### Sample collection and experimental design

Fresh testes-epididymides contained in scrotal sacs were collected from mature red Sokoto bucks slaughtered at a metropolitan abattoir in Makurdi, Benue state, Nigeria. Samples were individually packaged in polythene bags, placed on ice and transported in ice chest to the laboratory for further processing. On arrival, the testicles were stored at 5°C in refrigerator for 24, 48 h post-mortem and then processed (n=15 per each treatment group, respectively) while the other testicles (control) were immediately processed (n=15).

### Experimental procedure

In harvesting the testes and epididymides, the scrotal sacs were washed and cleaned with tap water. For thawing the samples, epididymides along with testes were allowed to warm to room temperature (35±2°C) for 15 min and then immersed into a water bath at 37°C for 5 min till the outer surface of the testes as well as epididymides were softened. Testicles were dissected away from the tunica vaginalis and other extraneous tissues during the warming period. A scalpel blade was used to make an incision from the dorsomedial aspect of the testis. The skin of the scrotum was reflected laterally, and the subcutaneous tissue and scrotal fascia were incised to expose the tunica vaginalis. The tunica albuginea was carefully removed from the testis. The epididymis was then carefully separated from the testis using the scalpel blade and thumb forceps. The testes and epididymides were excised, separated and weighed. The lengths of testes and epididymides were also measured using a ruler. The testes and the epididymides were also weighed on a sensitive balance scale (Ohaus^®^, USA).

Spermatozoa were collected from the caudal epididymides at room temperature by the incision method. Several incisions were made on the lower end of the epididymides to enable spermatozoa swim out into prewarmed (37°C) 5 ml, 2.9% sodium citrate buffer in a petri dish.

### Determination of sperm motility

Sperm motility from different treatment groups including control was evaluated as previously described [13]. Briefly, a drop (100 µl) of sperm sample was placed on a prewarmed, grease-free slide. A cover slip was put over the drop and examined under the microscope (40× magnification), and the percentage of sperm motility was determined.

### Determination of sperm concentration

Sperm concentration of each sample was determined using the improved Neubauer hemocytometer after appropriate dilution in 0.05% formol-saline as previously described [14].

### Determination of sperm viability

The viability of sperm cells was determined according to the method of Blom [15]. Briefly, a drop of sperm sample was mixed with a drop of eosin-nigrosin stain on a clean slide, and the mixture was allowed to stand for 3 min. Then, the smear was air dried and examined under the microscope (100× magnifications). Dead spermatozoa were stained either partially or completely pink or red, and live spermatozoa appeared colorless. 200 spermatozoa were randomly examined, and the percentage of live sperm cells was determined. The mean results were expressed as percent viable spermatozoa.

### Determination of acrosome integrity

The percent intact acrosome was determined according to the procedure previously described [16]. Briefly, a small (100 µl) drop of each sample was placed on a clean slide and a smear was made. The air-dried smears were fixed in Hancock’s solution for 15-20 min, washed and rinsed with distilled water. The slides were stained with Giemsa working solution overnight, then rinsed with tap water, air-dried and observed under the microscope (100× magnification). 200 spermatozoa were examined, and the percentage of intact acrosome was determined.

### Statistical analysis

Results were expressed as means and standard error of mean. Data were analyzed using one-way analysis of variance with software package (Graph Pad Prism for Windows; http://www.graphpad.com/scientific-software/instat). For analysis, p<0.05 was considered statistically significant.

## Results and Discussion

Testicular and epididymal parameters are reliable indices of assessing semen producing ability and in evaluation of breeding soundness of animals. This study describes the effect of storage time on testicular and epididymal biometry and cauda epididymal sperm values when abattoir-derived samples were processed immediately or stored at 5°C for 24 and 48 h, respectively. The mean values for testicular and epididymal lengths and weights in each treatment group (0, 24 and 48 h) are presented in Table-1. The effect of storage-time on epididymal sperm characteristics of red Sokoto bucks post-mortem are shown in Table-2. Sperm motility, concentration, viability and intact acrosome decreased significantly (p<0.05) from 0 to 48 h of storage.

**Table-1:** Mean (±SEM) testicular and epididymal values from red Sokoto bucks’ testes collected post-mortem, processed without storage and stored at 4°C for 24, and 48 h.

Parameter	Storage hours		

	0	24	48
Left testis weight (g)	56.82±0.62^a^	56.93±0.70^a^	56.99±0.65^a^
Right testis weight (g)	55.20±0.59^b^	55.75±0.64^b^	55.60±0.64^b^
Paired testes weights (g)	112.60±0.79	112.70±1.32	114.00±1.90
Left testis length (cm)	6.21±0.11	6.22±0.07	6.09±0.06
Right testis length (cm)	6.17±0.08	6.02±0.07	6.09±0.06
Paired testes length (cm)	12.52±0.19	12.21±0.12	11.87±0.14
Whole left epididymal weight (g)	6.94±0.04	6.75±0.10	7.02±0.05
Whole right epididymal weight (g)	6.80±0.04	6.64±0.09	6.85±0.04
Paired epididymal weights (g)	13.94±0.07	13.38±0.20	13.86±0.08
Whole left epididymal length (cm)	6.88±0.15	7.06±0.06	7.00±0.05
Whole right epididymal length (cm)	6.68±0.15	6.90±0.08	6.85±0.04
Paired epididymal lengths (cm)	13.54±0.21	14.01±0.10	13.85±0.08

Means with different superscripts in a column are significantly different (p<0.05). SEM=Standard error of mean

**Table-2:** Mean (±SEM) epididymal sperm parameters from red Sokoto bucks collected post-mortem, processed without storage, stored at 4°C for 24 and 48 h.

Parameter	Storage hours		

	0	24	48
Sperm motility (%)	79.93±0.66^a^	69.00±0.46^ab^	50.60±0.48^b^
Sperm concentration (×10^9^/ml)	2.86±0.08^a^	1.91±0.03^ab^	1.93±0.03^b^
Sperm viability (%)	83.40±0.58^a^	71.27±0.50^ab^	60.47±0.70^b^
Intact acrosome (%)	92.87±0.39	89.00±0.29	88.00±0.39

Means with different superscripts in a row are significantly different (p<0.05). SEM=Standard error of mean

The results of testicular and epididymal morphometry obtained in this study compare favorably with the previous values reported [17] for red Sokoto bucks raised in the savannah zone of Nigeria. The mean testis weight (81.7 g) and whole epididymal weight (14.93 g) reported by these authors were higher than the values in this study. These data were similar to the values of the testicular weights (51.79 and 52.23 g) and epididymal lengths (7.46 and 7.85 cm) earlier reported by Oyeyemi *et al*. [18].

Testicular weight and size vary with breed, age and time of the season. In this study, the left testis was larger than the right testis. The significant difference (p<0.05) observed in the ratio of left to right testes weights in the caprine bucks have also been reported in other studies [19]. Males with larger testes tend to produce more sperm [20]; hence a good measurement of testes weight, length and width would be a reliable index of predicting the sperm-producing capacity of bucks [21] and for selection of breeding males [22]. The findings in this study were similar to those reported by Mir *et al*. [23] who also did not observe significant differences in the mean testicular and epididymal values even after 48 h post-mortem.

The results of this study showed that cold storage up to 48 h reduced motility of sperm cells, sperm concentration, viability and intact acrosome (Table-2). In live animals, the cauda epididymides provide a suitable environment for the immature spermatozoa to become mature and acquire motility. Furthermore, studies have shown that spermatozoa recovered from cauda epididymides become motile only when they make contact with seminal fluid or media [24,25] and remain functional even hours after the death of an animal [12,26]. There are also reports that the handling conditions of testicles recovered post-mortem have some effect on the viability of spermatozoa [2].

The significant decrease in motility, sperm concentration, viability, and intact acrosome of epididymal spermatozoa recovered post-mortem and maintained under similar conditions have been reported in mice [27], ram [10,28], boar [5], bull [1], stallion [29], and camel [30]. Variation in quality of cauda epididymal sperm cells recovered post-mortem has been ascribed to handling conditions or species differences [31,32]. However, cold storage has the beneficial effect of keeping sperm cells viable for a longer period by slowing down the metabolic rate and degeneration of sperm cells [4,33].

Sperm concentration varies not only between animals but also between species of livestock [34]. The results in this study (Table-2) were higher than the values (0.61±0.05 × 10^9^/ml) earlier reported [35] but were within the range (1.12-4.66 × 10^9^/ml) previously reported [36] and were also in agreement with the finding of Hoseinzadeh-Sani *et al*. [37]. The results of this study revealed that viability of epididymal sperm cells declined in a storage time-dependent manner (Table-2). The values of this study were not in agreement with the findings of other researchers who recorded that only sperm motility and not live sperm cells were affected by storage temperature or post-mortem time [4,32]. On the other hand, data in this study are in accordance with the findings in bucks [38] and in red deer [39] that viability of sperm collected from the cauda epididymides post-mortem decreased progressively relative to the time of harvesting spermatozoa.

In this study, the percent intact acrosome decreased significantly (p<0.05) from 0 to 24 h, but not significantly (p>0.05) from 24 to 48 h (Table-2). The results of this study were in agreement with the findings of other scientists [23] who recorded 89.92% intact acrosome of cauda epididymal spermatozoa collected post-mortem in ram and stored up to 48 h at 4°C. These researchers observed that this recorded percent intact acrosome in addition to sperm motility (44.17%) and percent livability (78.96%) of cauda epididymal spermatozoa was of good quality and so indicate their high fertilizing potential. This observation is in agreement with an earlier finding [9].

## Conclusion

Despite the reduction in sperm characteristics during storage, epididymal sperm recovered post-mortem and stored at refrigeration temperature showed good motility, viability, and intact acrosome even after 24-48 h post-mortem. It is concluded that the testicles of red Sokoto bucks can be held at refrigeration temperature for up to 48 h until further processing. However, further research is required to determine if there are better methods that can be used to cryopreserve epididymal sperm recovered post-mortem. Furthermore, to increase the value of epididymal sperm retrieved postmortem, research protocols need to be developed that will utilize the harvested sperm cells in reproductive biotechnology.

## Authors’ Contributions

AHA planned and conducted the research work; TA helped in laboratory analysis. AIK helped with inputs in planning the research and surgical instruments. AHA, AIK, and TA carried out the statistical analysis and writing of the manuscript; AHA revised the manuscript. All authors read and approved the final manuscript.
